# In aged primary T cells, mitochondrial stress contributes to telomere attrition measured by a novel imaging flow cytometry assay

**DOI:** 10.1111/acel.12640

**Published:** 2017-08-19

**Authors:** Sharon Lesley Sanderson, Anna Katharina Simon

**Affiliations:** ^1^ Translational Immunology Laboratory NIHR BRC John Radcliffe Hospital Oxford OX3 9DU UK; ^2^ Kennedy Institute of Rheumatology University of Oxford Oxford OX3 7FY UK

**Keywords:** autophagy, ImageStream, lymphocytes, mitochondria, reactive oxygen species, telomere, T cells

## Abstract

The decline of the immune system with age known as immune senescence contributes to inefficient pathogen clearance and is a key risk factor for many aged‐related diseases. However, reversing or halting immune aging requires more knowledge about the cell biology of senescence in immune cells. Telomere shortening, low autophagy and mitochondrial dysfunction have been shown to underpin cell senescence. While autophagy has been found to control mitochondrial damage, no link has been made to telomere attrition. In contrast, mitochondrial stress can contribute to telomere attrition and vice versa. Whereas this link has been investigated in fibroblasts or cell lines, it is unclear whether this link exists in primary cells such as human lymphocytes and whether autophagy contributes to it. As traditional methods for measuring telomere length are low throughput or unsuitable for the analysis of cell subtypes within a mixed population of primary cells, we have developed a novel sensitive flow‐FISH assay using the imaging flow cytometer. Using this assay, we show a correlation between age and increased mitochondrial reactive oxygen species in CD8^+^ T‐cell subsets, but not with autophagy. Telomere shortening within the CD8^+^ subset could be prevented *in vitro* by treatment with a ROS scavenger. Our novel assay is a sensitive assay to measure relative telomere length in primary cells and has revealed ROS as a contributing factor to the decline in telomere length.

## Introduction

Telomeres are regions of repeating hexameric DNA sequences at the tips of chromosomes. Cells are unable to replicate the chromosomes ends resulting in a small loss of telomeric DNA at each cell division (Blackburn, 1991), which eventually will lead to replicative senescence. Cell senescence and biological aging is accompanied by mitochondrial dysfunction, known as the mitochondrial theory of aging (Harman, 1972). It is thought aging can occur as a result of accumulation of reactive oxygen species (ROS) followed by oxidative damage to molecules and DNA, especially in the oxidative phosphorylation complexes (Chistiakov *et al*., 2014). Once a threshold level of ROS is reached, this in turn leads to further mitochondrial dysfunction and cellular damage (Hekimi *et al*., 2011) including DNA damage at the telomeres to deteriorate genome stability (O'Sullivan & Karlseder, 2010). DNA damage kinases are activated, which induce a signalling cascade taking the cell into a transient proliferation arrest where they either undergo repair, apoptosis, or senescence (Kuilman *et al*., 2010). Autophagy turns over damaged organelles including mitochondria and thereby controls mitochondrial quality and ROS (Zhang *et al*., 2016). Decreasing levels of autophagy with age are thought to further enhance the mitochondrial vicious circle (Phadwal *et al*., 2012; Puleston *et al*., 2014).

Over a lifetime, naïve T cells divide very rarely and the little telomere loss that occurs is likely due to self‐renewing proliferation. However, naïve CD8^+^ T cells proliferate in response to a pathogen, and after the pathogen is cleared, they leave a small pool of memory cells, which form the basis of a more efficient expansion upon a second pathogenic encounter. Three types of CD8^+^ memory cells emerge from this: (i) the central memory T cell (T_CM_) is an early and least differentiated progenitor, with self‐renewal and homing potential to secondary lymphoid tissues; (ii) the effector memory T cell (T_EM_) with rapid effector function and homes to peripheral lymphoid tissues; (iii) the terminally differentiated and senescent effector memory T cells re‐expressing CD45RA T_EMRA_ with some effector function. Accordingly, proliferative potential decreases with progressive differentiation in these subsets T_N_ > T_CM_ > T_EM_ > T_EMRA_ (Mahnke *et al*., 2013).

The loss of telomeres in lymphocytes causes an age‐associated decline in immune function contributing to immune senescence (Iwama *et al*., 1998). Both the onset of cell autonomous senescence and diminishing naïve T‐cell output lead to impaired responses to new antigens, such as poor responses to the seasonal flu vaccine in the elderly (Boraschi & Italiani, 2014). Furthermore, replicative senescence due to telomere erosion in repeatedly stimulated T cells is seen in humans infected with a chronic infection, for example CMV (van de Berg *et al*., 2010; Riddell *et al*., 2015; Hoffmann *et al*., 2015). A link between shortened telomeres in the CD8^+^ T‐cell population and decreased vaccine efficacy has also been shown (Najarro *et al*., 2015). Autophagy is crucial in maintaining the CD8^+^ memory T‐cell population (Puleston *et al*., 2014; Xu *et al*., 2014). A link between declining autophagy levels with age and telomere shortening has not been investigated. The induction of telomerase activity can to some extent compensate for telomere loss in naïve and memory T cells (Greider, 1996). However, upon repeated stimulation, T cells lose the capacity to upregulate this enzyme leading eventually to telomere erosion and replicative senescence (Hodes *et al*., 2002).

A barrier to investigate the role of telomere attrition in the immune system is the difficulty in measuring telomere length at high throughput. Traditional methods such as terminal restriction fragmentation, polymerase chain reaction‐based techniques and single telomere length analysis require large cell numbers/DNA and cell sorting and are time‐consuming (Montpetit *et al*., 2014). While flow‐FISH techniques have been developed that allow cell identification by flow cytometry (Baerlocher *et al*., 2006), these assays have a low signal to noise ratio. We have developed a flow‐FISH technique for ImageStream (IS), an imaging flow cytometer (IS‐tel FISH). Flow‐FISH has been used successfully on the IS for aneuploidy measurement in primary blood mononuclear cells (PBMCs; Minderman *et al*., 2012), but has not been previously reported as a readout for telomere length or combined with surface markers.

Here, we validate the IS‐tel FISH in cell lines with known differences in telomere length, in PBMCs from different aged donors and thirdly in stimulated T lymphocytes in vitro. We show that this high‐throughput assay based on PNA spot counts is superior to flow‐FISH, which is based on intensity. We find telomere attrition occurs with increasing age alongside a decline in mitochondrial function and increasing ROS, but autophagy does not correlate with these mitochondrial parameters. We demonstrate a direct link between telomere attrition and ROS in CD8^+^ T cells activated *in vitro*, as telomere attrition can be prevented with a ROS scavenger.

## Results

### Validation of ImageStream telomere PNA FISH assay

**Figure 1:**
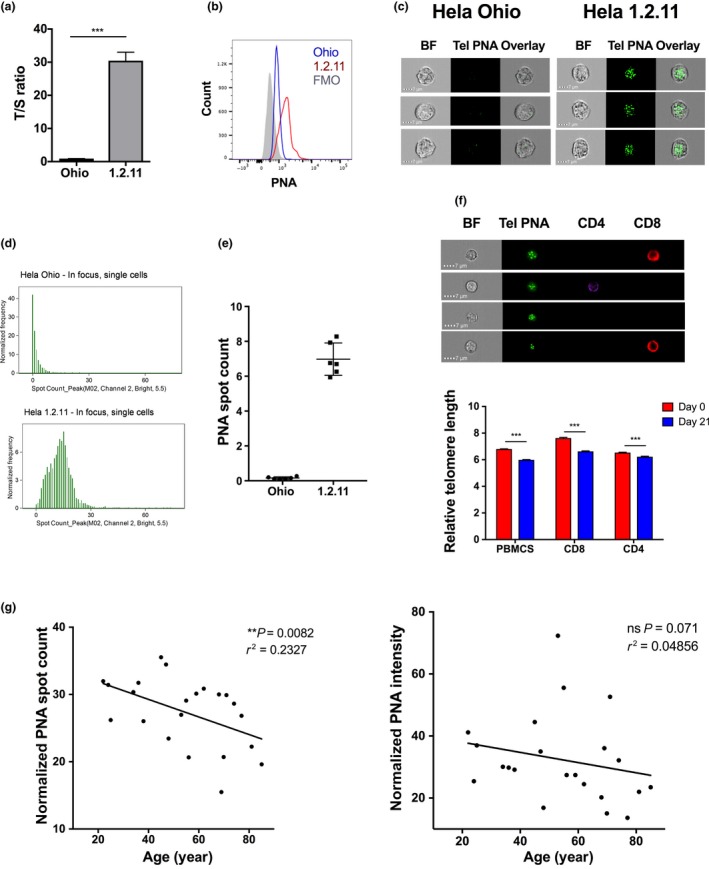
IS‐tel FISH assay detects telomere attrition in HeLa cell lines and human primary blood mononuclear cells (PBMCs). Telomere measurements of two HeLa cell lines by (a) qPCR (b) and flow‐FISH. (c) Brightfield (BF) and fluorescent images from ISXMKII (60× magnification) following IS‐tel FISH assay and (d) spot count frequency histograms of IS‐tel FISH analysis. (e) Average spot count frequency in HeLas performed in six independent IS‐tel FISH assays. (f) Brightfield and fluorescent images from ISXMII (60×) after IS‐tel FISH assay on human PBMCs cultured and stimulated for 0 and 21 days. Cells were stained with tel PNA–FITC, CD4‐PB and CD8–Cy5. Quantification of relative telomere length by spot count analysis. (g) Relative telomere length of PBMCs from healthy cohort quantified by spot count and intensity of tel PNA probe following IS‐tel‐FISH assay. Data shown as mean ± SEM, and stats show Mann–Whitney test (***P ≤ 0.001) or Spearman's correlation and *r* ^2^.

For optimization and validation of the IS‐tel FISH assay, HeLa OHIO and HeLa 1.2.11 with known differences in telomere length of ~3.4 and ~17 kb, respectively, were used (Thanasoula *et al*., 2010). These differences were detectable by qPCR (Fig. 1a). While flow‐FISH using a PNA telomere probe showed a significant overlap in intensity between the two HeLa cell types (Fig. 1b), analysis by spot count on the Image Stream XMII showed virtually no overlap in the number of spots (Fig. 1d,f; Fig. S1) demonstrating that it is a more robust assay. The assay was performed in HeLa cells in six independent experiments and showed high reproducibility with CV values of 42.91% for Ohio and 12.31% for 1.2.11 HeLa cells (Fig. 1e). A direct comparison between the novel IS‐tel FISH (spot count by IS) and flow‐FISH (intensity by flow cytometry) demonstrates that only IS‐tel FISH is able to detect a significant difference in telomere length with age in PBMCs using a limited number of just five donors (Fig. S1c). We tested the sensitivity of the assay and determined its suitability for use in human primary cells in freshly isolated peripheral mononuclear cells (PBMCs) before and after culture for 21 days (Chebel *et al*., 2009). To identify cellular subsets, antibodies to subset surface markers, that is CD4 and CD8, were included. Due to the heating step for DNA denaturation that precedes cell surface staining, only heat‐stable fluorochrome‐conjugated antibodies were found suitable. A small but significant reduction in telomere length could be detected in PBMCs, CD4^+^ and CD8^+^ T cells after 21 days in a proliferating culture in which PBMCs expanded fourfold, CD4^+^ T cells threefold and CD8^+^ T cells 23‐fold over that period (Fig. 1f; Fig. S1d).

### Telomere attrition in human PBMCs correlates with age by spot count but not intensity of PNA probe by ImageStream

To determine if telomere attrition can be detected in relation to donor age, relative telomere length was analysed in PBMCs from a small cohort of 22 healthy donors ranging from 22 to 85 years old. The significant correlation of age and telomere length observed (Fig. 1g) agrees with published data on telomere length (Iwama *et al*., 1998). Intensity of the tel PNA‐FITC probe measured by IS in the same experiment did not show this correlation, demonstrating that the imaging aspect of the telomere probe is more sensitive than intensity measurements using the ImageStream.

### Telomere attrition in CD8^+^ T cells compared to CD19^+^ B cells

**Figure 2:**
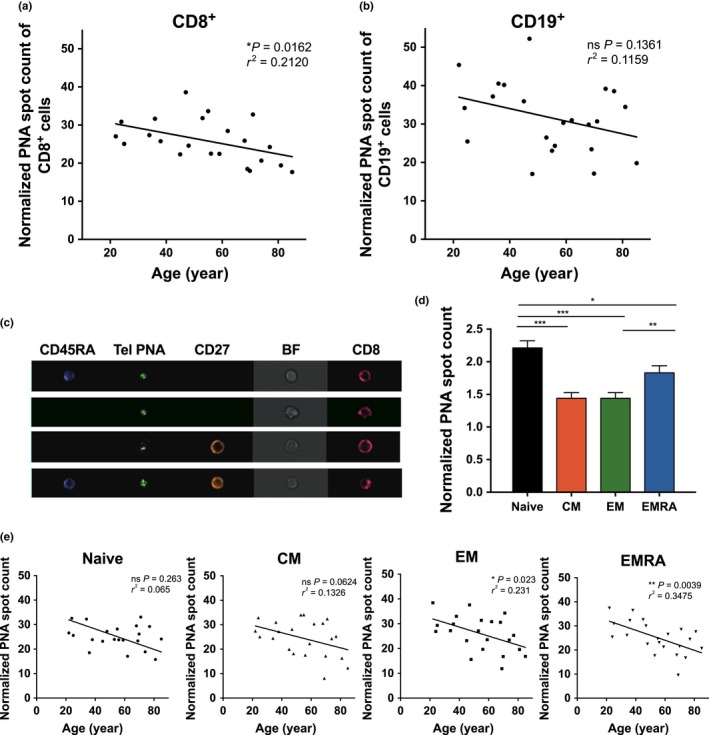
Inverse correlation between the donor age and telomere attrition in primary blood mononuclear cell (PBMC) CD8^+^ populations. PBMCs were analysed using the IS‐tel PNA FISH assay, staining for tel PNA‐FITC and CD19‐PB (panel 1) or tel PNA‐FITC, CD8‐Cy5, CD45RA–PB, CD27‐biotin and antibiotin Qdot 565 (panel 2) (a) CD8^+^ and (b) CD19^+^ cells were quantified for relative telomere length. (c) IS100 images (×40) after staining of CD8^+^ cells. (d) Mean relative telomere length quantification in the CD8^+^ populations and (e) correlated with age across the healthy donor cohort. Statistics show Spearman's correlation for correlation graphs and mean ± SEM and Wilcoxon paired *t*‐test for the bar chart.

We then compared relative telomere length in CD8^+^ (Fig. 2a) and CD19^+^ B‐cell populations (Fig. 2b), While a significant correlation of relative telomere length with donor age was found in CD8^+^ T cells, B cells only showed a insignificant correlation of telomere attrition with age, the average relative telomere length of CD8^+^ cells in the whole cohort being 25.87 compared to 30.37 in the CD19^+^ cells. This agrees with published data that telomere length is 15% higher in B compared to T cells (Martens *et al*., 2002). The rate of telomere shortening occurred at a faster but not statistically different rate in CD8^+^ T cells compared to B cells (slope = −0.1366 vs. −0.1642, respectively). Our data alongside published literature (Son *et al*., 2000) demonstrate that cell types in mixed populations have different telomere lengths; therefore, the inclusion of surface markers to identify cell populations is critical.

### Telomere length correlates with age in CD8^+^ memory T cells

To correlate telomere length with donor age in the four main CD8^+^ T‐cell subpopulations, we determined telomere length in each individual in our healthy cohort by staining for surface expression of CD27 and CD45RA molecules, that is naïve (CD27^+^ CD45RA^+^), T_EM_ (CD27^−^CD45RA^−^), T_CM_ (CD27^+^CD45RA^−^) and T_EMRA_ (CD27^−^CD45RA^+^) (Fig. 2c). When combining average data from the entire cohort, naïve CD8^+^ cells have the longest telomeres as expected, with reduced relative telomere length in T_CM_ and T_EM_ populations (Fig. 2d). We were able to demonstrate telomere attrition increased with age and correlates well with loss of proliferative potential known to occur in individual CD8^+^ populations (T_N_ > T_CM_ > T_EM_ > T_EMRA_), age having the largest effect on telomere length in the T_EMRA_ population (Fig. 2e).

### Mitochondrial health declines with age in CD8^+^ T cells

To correlate mitochondrial health and age in CD8^+^ T cells, we measured mitochondrial mass, membrane potential and reactive oxygen species in our cohort of young and old donors in the four main CD8^+^ T‐cell subsets using flow‐based assays (Wiley *et al*., 2014).

**Figure 3:**
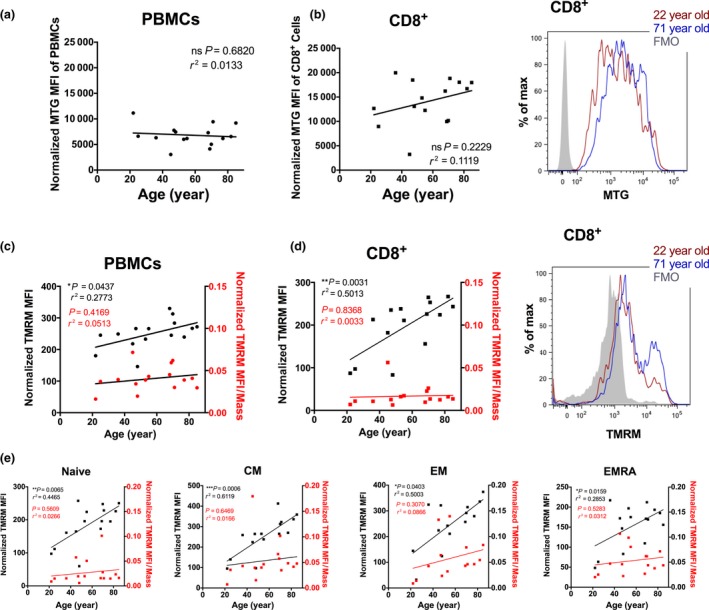
Donor age correlates with TMRM intensity but not with mitochondrial mass. Primary blood mononuclear cells (PBMCs) were quantified for mitochondrial mass by flow cytometry using a Mitotracker Green dye in (a) all live cells and (b) CD8^+^ subpopulation. Representative FACS histograms are shown from a young and old donor. PBMCs quantified for TMRM, which measures mitochondrial membrane potential by FACS using TMRM dye in (c) all live cells and (d) CD8^+^ subpopulation. Representative FACS histograms are shown from two donors. (e) Further CD8^+^ subpopulations were identified and quantified for TMRM expression. Statistics show Spearman's correlation.

MitoTracker Green (MTG) measures mitochondrial mass regardless of mitochondrial membrane potential (MMP). There was no correlation in whole PBMCs, the assay detection limit possibly being too low for this mixed population; however, the CD8^+^ T‐cell population exhibited a trend towards increased mitochondrial mass with age (Fig. 3a,b). No significant correlation to age was found in naïve CD8^+^ T cells, T_CM_, T_EM_ or T_EMRA_ populations (data not shown). However, the average mitochondrial mass in CD8^+^ T cells was twofold to threefold higher than in the mixed PBMC population particularly in the old donors (MFI = 6827 in PBMCs vs. MFI =14 095 in CD8^+^ T cells), indicating that age has more effect on mitochondrial mass in CD8^+^ T cells than other cell types contained in PBMCs (mostly CD4^+^ T cells and naïve B cells).

We next checked whether mitochondrial function was altered with age. We investigated this using the MMP sensitive dye tetramethylrhodamine methyl ester (TMRM), which accumulates in active mitochondria with intact membrane potentials. Upon loss of membrane potential, TMRM signal will dim; conversely increased TMRM indicates hyperpolarization. In both PBMCs and CD8^+^ populations, TMRM intensity per cell increased significantly with donor age (Fig. 3c,d). This pattern was mirrored in the CD8^+^ subpopulations with T_CM_ and T_EM_ populations displaying the most significant positive correlation of TMRM intensity with age (Fig. 3e). However, when normalized to mitochondrial mass, this increase was no longer significant (Fig. 3c–e in red).

### Mitochondrial ROS is increased with age in CD8^+^ T cells

Oxidative damage can cause telomere shortening, so lastly we measured mitochondrial ROS (mtROS) levels in CD8^+^ T‐cell subsets using MitoSOX Red. MitoSOX permeates live cells where it selectively targets mitochondria. It is rapidly oxidized by superoxide and becomes highly fluorescent upon binding to nucleic acid.

**Figure 4:**
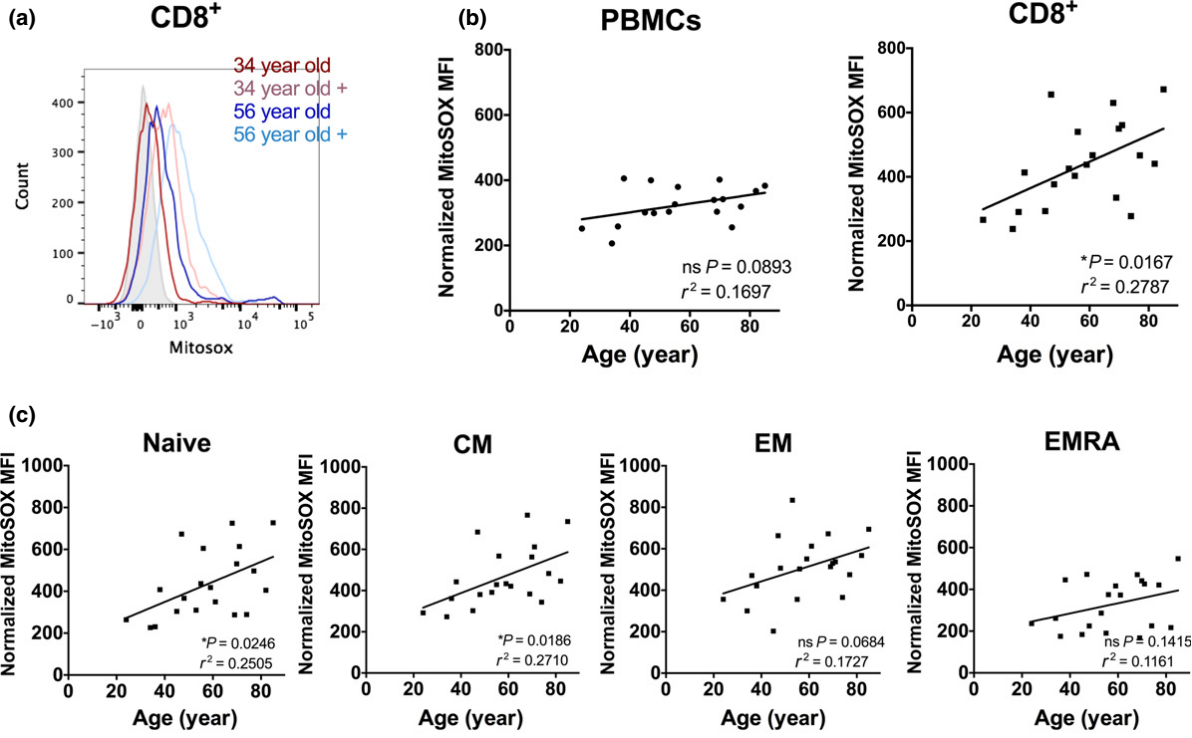
Mitochondrial ROS correlates with donor age in CD8^+^ populations. ROS levels were measured by flow cytometry using MitoSOX dye in (a) representative FACS histograms are shown of primary blood mononuclear cells (PBMCs) from two donors treated with 0.5 μg/mL antimycin A for 2 h as a positive control (indicated as +); (b) MitoSOX levels in PBMCs and CD8^+^ subpopulations. (c) Further CD8^+^ subpopulations were identified and quantified for MitoSOX expression. Statistics show Spearman's correlation and *r* ^2^.

As expected, antimycin A treatment induced mitochondrial ROS (Fig. 4a). Although no difference was seen in total PBMCs, there was a strong correlation of MitoSOX levels with age in CD8^+^ cells (Fig. 4b). We cannot exclude that this net increase in mtROS per cell is the result of a combination of increased mtROS per mitochondria along with an increase in the number of mitochondria. This same trend was seen in all CD8^+^ subpopulations, but this only reached significance in naive and T_CM_ populations (Fig. 4c). The same pattern of ROS levels as was found for basal levels was also seen with antimycin A treatment (data not shown).

Reactive oxygen species are known to cause oxidative damage to DNA by causing hydroxylation of guanine residues to 8‐hydroxy‐2′deoxyguanosine (8‐oxo‐dG). This can be measured by using an OxyDNA assay (Merck Millipore, Billerica, MA, USA). Interestingly, there was no correlation between oxidative DNA stress and age in neither PBMCs nor CD8^+^ T cells (Fig. S2a).

### NAC rescues telomere attrition in vitro in human CD8^+^ T cells

**Figure 5:**
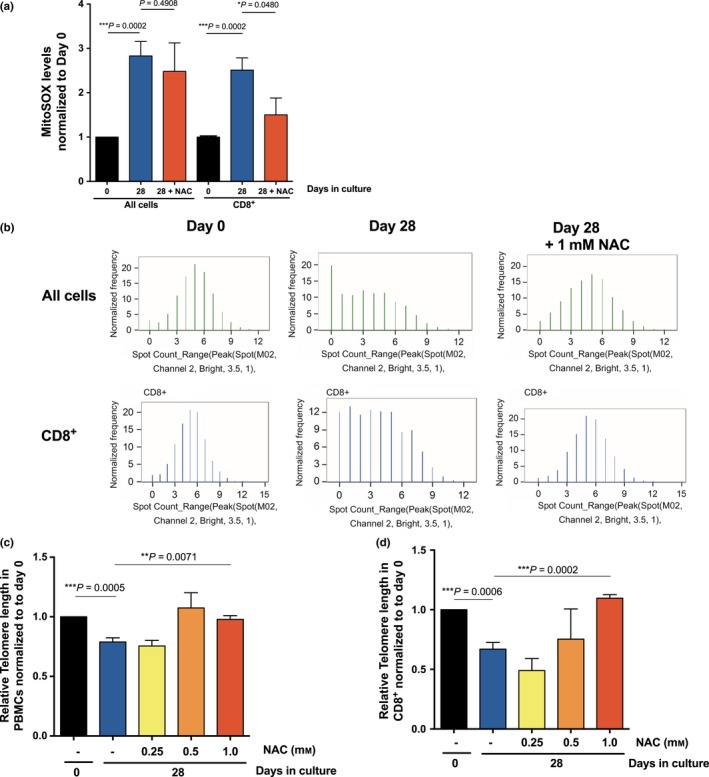
Telomere attrition in primary blood mononuclear cells (PBMCs) cultured for 28 days can be rescued by reactive oxygen scavenger NAC. (a) ROS levels were analysed using MitoSOX for all PBMCs and CD8^+^ cells cultured for 28 days ± 1 mm NAC. (b) Representative spot count frequency histograms from IS‐tel PNA FISH assay of PBMCs and CD8^+^ cells. Relative telomere length quantification of IS‐tel PNA FISH assay on (c) all PBMCs and (d) CD8^+^ cells, normalized to 1 for each donor. *n* = 4–8 donors from three independent experimental repeats. Data shown as mean ± SEM; stats show Mann–Whitney test.

In summary so far telomere length, TMRM intensity per cell and mitochondrial ROS correlate with age in human CD8^+^ T cells. We next investigated if the ROS scavenger *N*‐acetyl‐l‐cysteine (NAC) could rescue telomere attrition *in vitro* in PBMCs cultured over 28 days. Over the culture period, the cells showed significantly increased ROS levels and the addition of NAC was able to reduce mtROS significantly in the CD8^+^ population (Fig. 5a). Interestingly, we found that 28‐day NAC treatment rescued the telomere attrition as measured by average telomere spot count/cell by IS‐tel FISH (Fig. 5b) in PBMCs and CD8^+^ T cells (Fig. 5c,d).

### No correlation between autophagy and mitochondrial health in human CD8^+^ T cells

Mitochondrial health is controlled by mitophagy, a specific form of autophagy that degrades dysfunctional or damaged mitochondria. As expected, LC3‐II detected by flow cytometry was increased by treatment with a lysosomal inhibitor (BafA). We found the highest levels in T_EMRA_ cells (Fig. S2b). As opposed to other cell subsets, there was also a positive and significant correlation between ROS and relative basal autophagy levels in T_EMRA_ (data not shown). As we showed previously that autophagy‐deficient murine CD8^+^ memory T cells increase mitochondrial mass and reactive oxygen species (Puleston *et al*., 2014), it was of interest to examine this correlation in human CD8^+^ T cells over an age range. However, we found no significant correlation between autophagy levels and telomere length, MTG, TMRM or MitoSOX (Fig. S2b–d, respectively).

## Discussion

To analyse the link between mitochondrial health, autophagy and telomere length in human CD8^+^ T cells, we developed a sensitive telomere length assay, allowing for the identification of single cells in a mixed cell population. This assay was found to be superior to flow cytometry and was used to demonstrate for the first time telomere attrition in different CD8^+^ T‐cell populations in relation to age. While ROS levels were increased with age, mitochondrial mass was not. Surprisingly, autophagy levels did not correlate with these mitochondrial changes in this small cohort. However, scavenging ROS prevented telomere attrition in repeatedly stimulated CD8^+^ T cells *in vitro* demonstrating a causal relationship. Finally on average over the entire cohort, the conventional memory populations showed shortest telomeres with increased mitochondrial ROS in line with our hypothesis of a link in human primary lymphocytes.

We have developed a novel single cell assay to measure telomere length and multiparameters simultaneously. The IS‐FISH approach enables the evaluation of 100 000s of cells in suspension, and the analysis can be automated and standardized diminishing operator bias. The high cell number throughput of IS‐FISH improves the detection of rare events compared to conventional FISH.

The analysis of this assay calculates average tel PNA spot count/cell. While unlikely that telomeres from every chromosome in the cell are detected using this method, it is rather telomeres over a certain length, the threshold being determined by the resolution of the IS. However, due to the large number of cells analysed, we have demonstrated that this gives a robust readout of the average relative telomere length. Spot count was superior to alternative analysis methods such as relative spot count intensity and peak measurements.

Our assay readout is relative mean telomere content normalized to an internal standard. However, this could be further improved in future to include a human reference sample, with known telomere length in every experiment to calculate actual telomere length rather than relative such as used for flow‐FISH (Baerlocher *et al*., 2006).

Only one earlier pioneering study has combined *in situ* hybridization with IS to detect aneuploidy (Minderman *et al*., 2012). Together with the addition of surface markers introduced here, this is now an extremely versatile technique that could be applied to rare cell populations such as stem cells. It also has the potential to be extended to other FISH probes that detect chromosomal abnormalities in human mixed and rare cell populations at high throughput without cell sorting.

Several studies report differential telomere length in subsets of CD8^+^ T cells. CD8^+^ T cells lose expression of costimulatory receptors CD28 and CD27 with age and this population has shorter telomeres than CD28^+^CD27^+^ T cells (Plunkett *et al*., 2007; Lin *et al*., 2010). More recently, a study demonstrated a significant difference between telomere length in CD8^+^ naïve, T_CM_, T_EM_ and T_EMRA_ (based on CD27 and CD45RA expression) in young (18‐ to 40‐year‐olds) and old (65 years of age and over) donors. It also showed that naïve CD8^+^ T cells had longer telomeres and T_EM_ population the shortest (Riddell *et al*., 2015). Results from our sensitive assay concur with these recent data.

Interestingly, CD8^+^ T_EMRA_ cells were found not to have the shortest telomeres, as shown before (Riddell *et al*., 2015). On the other hand, most studies agree that proliferative potential of T_EMRA_ is the lowest of all memory cells (Mahnke *et al*., 2013), which one would not predict from the longer telomeres. Our collaborators proposed that inhibition of p38 signalling in the T_EMRA_ population increases proliferation, telomerase activity and mitochondrial biogenesis, with the extra energy provided by autophagy (Henson *et al*., 2014). Correspondingly, the only positive correlation we could find between autophagy levels and ROS was in the T_EMRA_ population.

Although significance was reached in most assays, it should be noted that these results are based on an extremely small sample size. The second major caveat is that we do not know the CMV status of our donors. CMV serotype‐positive individuals have been reported by several studies to have shorter telomeres than matched negative individuals (Spyridopoulos *et al*., 2009) and (Hoffmann *et al*., 2015). However, even without taking into account CMV status, our data show significant telomere attrition with age. Moreover, other issues with donor health status can skew data. For example, it has been reported that cardiac disease can impact on telomere length. Similar to ours, a study by Hoffmann *et al*. shows that healthy donors have longer telomeres in T_EMRA_ populations compared to T_EM_; however, this was reversed in CMV^+^ donors with myocardial infarction (Hoffmann *et al*., 2015). To the best of our knowledge, our donors, however, did not report any severe cardiac abnormalities beyond what is expected in those of an advanced age (>75 years).

The two main theories of aging are (i) the accumulation of genotoxic stress provoked by the progressive loss of telomeres leading to replicative stress and cell death, and (ii) the increasing loss of mitochondrial function (Passos *et al*., 2007). These two theories have also been molecularly linked by a series of studies (reviewed in Correia‐Melo *et al.,* 2014). Indeed, a recent study by DePinho *et al*. shows that telomerase knockout mice with severe telomere shortening in later generations decrease mitochondrial biogenesis due to p53‐mediated repression of mitochondrial PGC1α and β genes (Sahin *et al*., 2011).

On the other hand, a series of studies demonstrate that telomere attrition is dependent on mitochondrial function (von Zglinicki, 2002, reviewed in Passos *et al*., 2007) and significant correlations have been found between mitochondrial mass and telomere length in PBMCs (Wiley *et al*., 2014). The intensity of dosage of oxidative stress determines where the DNA damage occurs, either in the bulk of the genome or at the telomeres. Low‐level chronic oxidative stress mainly generates single‐strand breaks and telomeres acquire such breaks at a faster rate firstly due to their guanine‐rich sequences, secondly their repair machinery is less efficient, and lastly oxidative stress interferes with telomere maintenance (Passos *et al*., 2007). If oxidative stress is diminished using free radical scavengers, telomere attrition is diminished (Cui *et al*., 2012). Most of these studies have been performed in cell lines. Here for the first time, we confirm that scavenging ROS halts telomere attrition in cultured primary T lymphocytes. While we cannot exclude that this an artefact due to the high oxygen concentration in tissue culture, our careful correlative analysis of different T‐cell subsets from healthy human donors shows a strong link between age and telomere length, mitochondrial ROS and health suggesting that oxidative damage contributing to telomere attrition may also be true under physiological conditions in humans.

The role of autophagy in telomere attrition had not been addressed before. However, our data fail to show a correlation between autophagy levels and telomeres or with reactive oxygen species. This was a surprising result as total tissue‐specific loss of autophagy leads to increased reactive species in every hematopoietic cell type we have examined to date (Stranks *et al*., 2015; Puleston *et al*., 2014) and premature aging of the immune system (Zhang *et al*., 2016). Our novel assay is not able to measure telomere attrition in mouse cells preventing us from examining telomere attrition in autophagy‐deficient primary immune cells.

In summary, this study indicates that reactive species that accumulate with age contribute to telomere shortening in primary memory T lymphocytes.

## Experimental procedures

### Cell lines

HeLa 1.2.11 and HeLa OHIO adherent cells were grown in Dulbecco's modified Eagle's medium (Sigma‐Aldrich, St. Louis, MO, USA) supplemented with 10% foetal bovine serum, penicillin and streptomycin.

### PBMC extraction

30–50 mL of blood was obtained in heparinized tubes with the approval of a local ethics committee (REC reference 11/H0504/1) from healthy donors aged between 22 and 85 years old, of which 60% were males. All donors reported to be healthy and specifically had no known coronary heart disease, HIV or hepatitis. PBMCs were separated using Lymphoprep (Axis‐Shield, Dundee, Scotland) and frozen in aliquots.

### Telomere qPCR

DNA was extracted using Qiagen DNeasy blood and tissue kit (Thermo Fisher Scientific, Waltham, MA, USA) and 20 ng used for qPCR. qPCR was performed based on a method by Cawthon (Cawthon, 2009) to determine the relative telomere length (T) across all chromosomes relative to the amount of a single copy reference gene (S), in this case 36B4.

Primers sequences and the concentrations used at were as follows: TelF (5′‐GGTTTTTGAGGGTGAGGGTGAGGGTGAGGGTGAGGGT‐3′ at 300 nm), TelR (5′‐TCCCGACTATCCCTATCCCTATCCCTATCCCTATCCCTA‐3′ at 900 nm), 36B4F (5′‐CAGCAAGTGGGAAGGTGTAATCC‐3′ at 300 nm), 36B4R (5′‐CCCATTCTATCATCAACGGGTACAA‐3′ at 600 nm).

PerfeCTa SYBR Green FastMix (Quanta Biosciences, Beverly, MA, USA) was used and qPCR performed using a Bio‐Rad CFX96. Cycling conditions for tel qPCR were 95 °C for 10 min; 25 cycles of 95 °C for 15 s, 54 °C for 60 s and 72 °C for 30 s. The cycling conditions for 34B4 were 95 °C for 10 min; 30 cycles of 95 °C for 15 s, 58 °C for 60 s and 72 °C for 30 s.

The relative gene concentration was calculated from CT values using a standard curve. An average of triplicate measurements was used to calculate the T/S ratio.

### ImageStream Telomere PNA FISH (IS‐tel FISH) assay

5 × 10^6^ cells were stained for surface markers, if required, using the following antibodies: biotin antihuman CD27 (O323), PB antihuman CD19 (HIB19), PB antihuman CD45RA (HI100), IgM antihuman cy5 (Bioss, Boston, MA, USA), cy5 antihuman CD8 (RTF8, Southern Biotech, Birmingham, AL, USA), qdot 705 antihuman CD38 (HIT2, Thermo Fisher Scientific) and Qdot 565 streptavidin conjugate (Thermo Fisher Scientific).

Surface proteins were cross‐linked by adding 31 μL of fresh 12.5 mm BS^3^ (Thermo Scientific) to each sample, including single‐colour compensation controls, and incubating at 4 °C for 30 min in dark; 9 μL/well quenching buffer (1 m Tris, pH 7.5) was added followed by incubation for a further 15 min at RT shaking. The buffer was stained for PNA‐FITC following manufacturer's instructions in the PNA Telomere Kit for FACS (DAKO, Santa Clara, CA, USA). Briefly, it was washed with cold PBS + 5% FCS, transferred to 0.5‐mL Eppendorf tubes and incubated with 100 μL of PNA‐FITC or 100 μL of hybridization buffer at 80 °C for 10 min. Tubes were left overnight at room temperature before washing twice with wash solution for 10 min at 40 °C. The solution was resuspended in PBS + 5% FCS. Samples were ran on ImageStream 100 or ImageStream Mark II (Merck Millipore); 30 000 events were saved from samples and 500 positive events from compensation controls.

### ImageStream Telomere PNA FISH assay analysis

Analysis was performed using ideas ^®^ V6.2. Single‐colour controls were used to create a compensation matrix that was applied to each file to compensate for spectral spillover. The resulting compensated files were analysed using algorithms available in ideas ^®^. In focus, cells were identified using gradient RMS of the brightfield image, and those with a higher gradient RMS are more in focus. Single cells were then identified from debris and cell clusters in the in‐focus population using a bivariate plot of aspect ratio vs. area of the brightfield channel. Subpopulations can be identified based on a positive intensity signal from each channel. Cells of interest are then analysed for tel PNA expression. This can be done by geometric mean of the fluorescent intensity or by using a Spot count Wizard. Truth populations are selected of at least 30 cells with high or low (but not none) tel PNA spots in the images. The more accurate this selection, the better the mask that is created. The wizard uses Fisher's discriminant ratio (Rd) to determine the spot count Feature/Mask by finding the best statistical separation (largest Rd) based on the ‘truth’ populations. If the mask is not robust, then the ‘truth’ populations may need refining. Once an appropriate mask and feature has been created, then the template can be saved and applied to the other data analysis files (Fig. S1).

For telomere measurements in the cohort, it was necessary to normalize data to take into account that samples were not processed and analysed at the same time. Samples were ran in batches, and in each case, HeLa 1.2.11‐stained and unstained cells were also ran as a normalization control. Each experiment used HeLa cells that had been frozen at the same time to ensure there was no variation in telomere length between controls. Spot count and intensity was normalized, with stained HeLa 1.2.11 set to 100 and unstained to 0. HeLa 1.2.11 was chosen as these cells had longer telomeres than the human donors allowing for a relative scale between 0 and 100 to be used.

### Long‐term PBMC culture

PBMCs were cultured in RPMI‐1640 (Sigma‐Aldrich) supplemented with 10% foetal bovine serum, penicillin and streptomycin. At days 0 and 14, PHA‐L (Merck Millipore) was added at 10 ug/mL. On day 1 and every third day, 50 U/mL IL2 (Biolegend, San Diego, CA, USA) ± NAC (Sigma‐Aldrich) was added. Cells were frozen at the end of the culture period for use in subsequent assays alongside cells frozen at day 0 from the same donor.

### Flow cytometry

Flow cytometry experiments were performed on Fortessa or LSRII instruments (Becton Dickinson, Franklin Lakes, NJ, USA), and PBMCs from the healthy donor cohort were stained for several functional assays where cell number allowed. Firstly, cells were stained using Zombie NIR fixable viability kit (Biolegend), and dead cells were excluded from subsequent analysis. Surface staining was performed using the following antibodies: PE/cy7 antihuman CD8 (SK1), APC antihuman CD14 (HCD14), BV421 antihuman CD45RA (HI100), PERCP antihuman CD19 (SJ25C1), PE antihuman CD27 (M‐T271), FITC antihuman CD27 (O323). All antibodies were obtained from Biolegend, unless indicated otherwise.

8‐oxo‐dG detection was performed an OxyDNA assay kit (Merck Millipore) according to instructions. Prior to staining, cells were incubated with or without 50 μm H_2_0_2_ in PBS with 5% FCS for 1 h at 37 °C. After live–dead and surface marker staining, cells were fixed using Foxp3 buffer (eBioscience, San Diego, CA, USA). Cells were then washed in permeabilization buffer (eBioscience) and incubated in 8‐oxo FITC conjugate diluted in the wash buffer contained in the kit for 1 h at room temperature.

Mitochondrial stains were performed after live–dead and surface marker staining by incubating cells at 37 °C for 15 min with 5 μm MitoSOX red, 37 °C for 25 min with 150 nm MitoTracker green or at 37 °C for 15 min with 100 nm tetramethylrhodamine methyl ester (TMRM) (all from Thermo Fisher Scientific).

A FlowCellect Autophagy LC3 Antibody‐based Assay Kit (Merck Millipore) was used to detect LC3‐II. PBMCs were incubated in RPMI medium supplemented with 10% foetal bovine serum, penicillin and streptomycin with or without 10 nm bafilomycin A1 (Sigma‐Aldrich) for 2 h at 37 °C. Cells were stained for LC3‐II following live–dead and cell surface antibody staining according to the manufacturer's instructions using a LC3‐FITC‐conjugated antibody. The kit included a step to wash out unbound cytosolic LC3‐I leaving only membrane‐bound LC3‐II, which was detected by flow cytometry.

The cohort samples were stained in batches for each assay. To avoid minor variations in experiments performed on different days, the results were normalized to data from a PBMC control sample that was included each time. FACS data were analysed using flowjo V10.0.8.

## Funding

This work was funded by the NIHR Biomedical Research Center (BRC), the Wellcome Trust (103830/Z/14/Z) and the MRC Human Immunology Unit.

## Author contributions

SS and KS conceived the project. SS conducted the experiments and interpreted the data. SS and KS wrote the manuscript.

## Conflict of interest

The authors have no conflict of interest to declare.

## Supporting information


**Fig. S1**. Gating strategy used to measure relative telomere length in CD8^+^ and CD4^+^ PBMCs and comparison of flow‐FISH and IS‐tel FISH assays.

**Fig. S2**. In CD8^+^ T cells oxidative DNA damage does not correlate with age, also autophagy levels do not correlate with telomere length or mitochondrial stress.
